# Magnetocardiography at rest predicts cardiac death in patients with acute chest pain

**DOI:** 10.3389/fcvm.2023.1258890

**Published:** 2023-12-14

**Authors:** N. Wessel, J. S. Kim, B. Y. Joung, Y. G. Ko, D. Dischl, A. Gapelyuk, Y. H. Lee, K. W. Kim, J. W. Park, U. Landmesser

**Affiliations:** ^1^ Department of Human Medicine, MSB Medical School Berlin GmbH, Humboldt-Universität zu Berlin, Berlin, Germany; ^2^ Department of Physics, Humboldt Universität zu Berlin, Berlin, Germany; ^3^ Division of Cardiology, Department of Internal Medicine, Severance Cardiovascular Hospital, Yonsei University College of Medicine, Seoul, Republic of Korea; ^4^ Deutsches Herzzentrum der Charité, Department of Cardiology, Angiology and Intensive Care Medicine, Berlin, Germany; ^5^ Charité - Universitätsmedizin Berlin, Corporate Member of Freie Universität Berlin and Humboldt-Universität zu Berlin, Berlin, Germany; ^6^ Center for Biosignals, KRISS Korea Research Institute of Standards and Science, Daejeon, Republic of Korea

**Keywords:** sudden cardiac death, magnetocardiography, acute chest pain, Kaplan–Meier estimator, Cox regression model

## Abstract

**Introduction:**

Sudden cardiac arrest is a major cause of morbidity and mortality worldwide and remains a major public health problem for which better non-invasive prediction tools are needed. Primary preventive therapies, such as implantable cardioverter defibrillators, are not personalized and not predictive. Most of these devices do not deliver life-saving therapy during their lifetime. The individual relationship between fatal arrhythmias and cardiac function abnormalities in predicting cardiac death risk has rarely been explored.

**Methods:**

We retrospectively analyzed the measurements at rest for 191 patients with acute chest pain (ACP) magnetocardiographically. Our recently introduced analyses are able to detect inhomogeneities of the depolarization and repolarization. Moreover, electrically silent phenomena—intracellular ionic currents as well as vortex currents—can be measured and quantified. All included ACP patients were recruited in 2009 at Yonsei University Hospital and were followed up until 2022.

**Results:**

During half of the follow-up period (6.5 years), 11 patients died. Out of all the included nine clinical, eight magnetocardiographical, and nine newly introduced magnetoionographical parameters we tested in this study, three parameters revealed themselves to be outstanding at predicting death: heart rate-corrected QT (QTc) prolongation, depression of repolarization current I_Kr_ + I_Ks_, and serum creatinine (all significant in Cox regression, *p* < 0.05). They clearly predicted cardiac death over the 6.5 years duration (sensitivity 90.9%, specificity 85.6%, negative predictive accuracy 99.4%). Cardiac death risk was more than ninefold higher in patients with low repolarization reserve and QTc prolongation in comparison with the remaining patients with ACP (*p* < 0.001). The non-parametric Kaplan–Meier statistics estimated significantly lower survival functions from their lifetime data (*p* < 0.001).

**Discussion:**

To the best of our knowledge, these are the first data linking magnetocardiographical and magnetoionographical parameters and subsequent significant fatal events in people, suggesting structural and functional components to clinical life-threatening ventricular arrhythmogenesis. The findings support investigation of new prevention strategies and herald those new non-invasive techniques as complementary risk stratification tools.

## Introduction

Acute chest pain (ACP) and acute coronary syndrome (ACS) refer to a number of cardiac conditions associated with an unmediated reduction in blood flow to the heart (1). One such condition may be a heart attack, in which there is pathological change in the heart tissue. ACS is a medical emergency that requires rapid diagnosis and treatment; the patients recruited in this study at Yonsei University Hospital received personalized therapy—hence the mortality of such patients is low in comparison with other investigations (2).

Coronary artery disease (CAD) is an inflammatory process that initially leads to non-obstructive and eventually obstructive atherosclerotic plaques. As CAD is characterized by atherosclerosis and may be asymptomatic, ACP in nearly all cases is associated with symptoms, such as unstable angina, regardless of the presence of CAD (3). A life-threatening ischemia occurs when these atherosclerotic plaques of the coronary arteries become severely obstructive. Both obstructive and non-obstructive stenoses can lead to acute circulatory disturbances of the heart. Hence, they can cause myocardial infarction, acute plaque rupture, and thrombosis. Clinical scores such as age, gender, cardiovascular risk factors, electrocardiogram (ECG) changes, or enzymes can be used to determine statistically (but not individually) whether patients have a low, intermediate, or high pre-test probability of CAD (1). Individually, chest pain, angina-like symptoms, shortness of breath on exertion, and left arm pain are indicative of potential circulatory problems. In patients with known coronary syndromes or a high pre-test probability of CAD, invasive coronary angiography is performed. Electrocardiographic exercise stress testing followed by potential non-invasive imaging [coronary computed tomography angiography (CTA)] is warranted in patients with clinical symptoms and suspected cardiac cause (4).

Stress electrocardiography is the standard examination procedure in community practices and hospitals and the first functional test in suspected CAD (5). However, the test protocol can be interrupted or extended based on the fatigue level or, if the patient develops cardiac symptoms, significant ECG changes, or other risk features. However, a meta-analysis of 24,074 patients in 147 studies found that the ECG stress test for the detection of CAD had a sensitivity of only 68% and a specificity of 77% (6).

Nearly 850,000 patients underwent diagnostic cardiac catheterization in Germany in 2019, but only about 50% required interventional or cardiac surgery consequences (7). Whether a non-invasive diagnostic test is appropriate is determined by the CAD pre-test probability. Statistically, these tests are only meaningful in patients with values between 15% and 85%. Despite widespread availability, decades of experience, no radiation exposure, low financial expense, and demonstrated clinical usefulness of exercise ECG, the disadvantages are serious.

The 2019 German national guideline on chronic CAD (8) classifies stress ECG only as a second-line diagnostic method, when imaging modalities are not available locally and the pre-test probability is between 15% and 30%. By contrast, diagnostic imaging such as CTA, stress echocardiography, myocardial perfusion SPECT, or stress MRI are now recommended by national as well as international guidelines when chronic stable CAD is suspected. This is because these methods not only have a higher diagnostic sensitivity but are also possible to use in non-ergometric patients.

CTA is recommended for pre-test probability between 15% and 50% and allows anatomically accurate morphological visualization of the coronary arteries. The sensitivity is 95%–99%, which is significantly better than its specificity (64%–83%). Some disadvantages are possible contrast agent allergy (iodine), limited contrast agent amount, and radiation exposure. In addition, CTA is not covered by the statutory health insurance, and must be paid by the patients themselves.

Sudden cardiac death (SCD) is defined as an unexpected death or cardiac arrest that occurs rapidly, usually because of fatal ventricular arrhythmias in the setting of underlying CAD (9). Despite major advances in CAD treatment and the use of implantable cardioverter defibrillators (ICDs) to prevent SCD, SCD remains a major public health problem, estimated to account for up to 20% of all deaths. Approximately 80% of individuals who suffer SCD have CAD (10). Another group of high-risk patients for SCD includes those with hereditary ion channel or myocardial defects, such as a long QT syndrome (LQTS) or short QT syndrome (SQTS), hypertrophic cardiomyopathy (HCM), and arrhythmogenic right ventricular dysplasia (ARVD) (11). All risk groups for SCD most likely share a common feature of impaired intracellular calcium homeostasis (12). The main causes of death in heart failure patients, declining cardiac pump function and arrhythmias, have both been linked to the disrupted Ca^2+^ homeostasis in cardiac muscle cells (13).

Non-invasive magnetography is widely used in the field of medicine, in particular for the diagnosis of a dysfunction of the brain or heart tissue (14). Magnetocardiography (MCG) is a non-contact, non-invasive, radiation-free, and non-contrast method that allows the recording of magnetic fields generated by the electrical activity of the heart (15). Although electrocardiography (ECG) and MCG provide information about the same electrical activity of the heart, MCG offers significant advantages. The magnetic fields of the heart remain unaffected by variations in the conductivity of body tissues or fluids, showing no attenuation or distortion (16). Several clinical studies have already shown the superiority of MCG over ECG in detecting myocardial infarction both at rest and during exercise [(15) and references therein]. MCG is useful in the examination of heart dysfunction, particularly for CAD (17, 18). The aim of this study was to prove that MCG together with recently developed magnetoionographical features is able to detect the individual risk for cardiac death.

## Methods

### Patients

A total of 245 patients who came to Yonsei University Hospital and had undertaken MCGs for acute chest pain between November 2008 and July 2009 were included in this study. We retrospectively analyzed the measurements of 191 of these patients, where MCG recordings and coronary angiography or stress test were available. All patients were recruited in 2009 at Yonsei University Hospital and were followed up until 2022. The patients were followed up at 1-month, 3-month, and 6-month intervals life long after discharge from the clinic. All long-term clinical data were derived from the Severance Hospital Information System. This study was approved by the local ethics committee and informed consent was obtained from each subject.

### Magnetocardiography

In cardiac diseases, the inflammation of damaged areas and the disruption of the orderly physiological sequence of electrical excitation processes lead to a change in the electrochemical properties of the heart muscle cells (electrical remodeling). This leads to an inhomogeneity of the depolarization and repolarization processes, which can be detected with an MCG (19, 20). An MCG can measure tangential currents that occur at the boundary between damaged and healthy tissue, which the ECG cannot detect. In addition, an MCG is able to measure electrically silent phenomena—intracellular ionic currents as well as vortex currents. The recently introduced methodology for the detection and characterization of individual ionic currents on a cellular level is called magnetoionography [MIG (21)]. With MIG, pathologies are inferred from a generalized human muscle cell physiology, considering medical knowledge, in particular knowledge about ion fluxes. MCG is an effective tool for detecting electrical restructuring, as has been demonstrated in various animal experiments and clinical studies (17, 22).

The MCG and MIG parameters were calculated for different cardiocycle intervals: 1: QRS, 2: QRS_end_–T_beg_, 3: T_beg_–T_end_, 4: QRS_end_–T_end_. For example, RMS_1_ means average root mean square (RMS) of all 64 channels during QRS. With regard to the MIG parameters, MI stands for “monopolarity index” and DI for “dipolarity index”, both quantifying the topologic polarity of the cardiac magnetic field maps. Furthermore, the MI and DI parameters are weighted by RMS in the respective intervals to quantify the influence of field strength. Summarizing, these algorithms are derived from combined time-specific (1-QRS to 4-QRS_end_-T_end_) information of cardiac magnetic field maps concerning polarity (MI, DI, and the logarithm of MI for calculation purposes), current density flow, and strength (RMS). Ca^2+^  uptake velocity is defined as the maximum Ca^2+^ transient curve decrease velocity (pT/sec) in the time period T_max_–T_end_.

The MCG recordings were performed at high resolution using a 64-channel biomagnetometer from KRISS in a magnetically shielded room at the Severance Hospital (Bio-Signal Research Center, KRISS, Daejeon, Korea). This MCG system uses superconducting quantum interference sensors with double relaxation oscillation. The average noise spectral density of the whole system in the magnetically shielded room was 10 fT/Hz at 1 Hz and 5 fT/Hz above 100 Hz. The system is equipped with planar first-order superconducting gradiometers that measure the tangential components of the cardiomagnetic fields. A high-pass filter of 0.5 Hz, a low-pass filter of 1.6 kHz, and a 60 Hz notch filter were used for recording. The magnetocardiographic recordings were performed under resting conditions (30 s recording, 500 Hz) after the patient had rested on the bed for at least 2 min. After recording, the signals were baseline corrected, digitally filtered, and averaged to increase the signal-to-noise ratio. Finally, the data were averaged and centered on the peak of the R-wave.

### Statistical analysis

Stepwise Cox regression analysis was performed in SPSS (v.27). It allows us to adjust for several variables. A *p*-value below 0.05 was considered as significant. Linear discriminant analysis (LDA) (23) was used to obtain a classification rule to separate the survivors from the deceased. The robustness of the resulting classification was checked by means of a leave-one-out cross-validation, which has been proved to minimize bias and mean squared error in an LDA (24). The non-parametric Mann–Whitney *U* test was deployed to assess if there is a significant difference in the clinical characteristics between the two groups (25). And the Kaplan–Meier analysis was used to assess the relation between the different predicted group memberships and survival (years) (26).

## Results

A total of 191 patients with ACP [age 62.1 ± 10.9 years; 114 men (59.7%); 77 women (40.3%)] were recruited between November 2008 and July 2009 at Yonsei University Severance Hospital. The patients were admitted to the hospital with chest pain without ST segment elevation in electrocardiography. The clinical diagnosis was based on typical chest pain, cardiac enzyme [plasma creatine kinase (CK) cardiac isoenzyme level], and echocardiographic findings. Coronary angiography was performed and left ventricular ejection fraction (LVEF) with echocardiography was obtained in 176 patients and lead to CAD diagnosis in 121 patients (single vessel disease *n* = 39, two-vessel disease *n* = 42, three-vessel disease *n* = 40), whereas in 55 patients with ACP, no CAD was diagnosed.

All patients were followed up until 2022. During half of the follow-up period (6.5 years), 11 patients died. The clinical characteristics of the survivors vs. the deceased at 6.5 years are provided in Table 1.

**Table 1:** Clinical characteristics of the 180 patients with ACP who survived the 6.5 years follow-up vs. the 11 deceased.

Parameter	Survivors (*n* = 180)	Cardiac death (*n* = 11)	*p*-value
Age	62.0 ± 11.1	64.4 ± 10.2	n.s.
Gender	110 (F)	4 (F)	n.s.
BMI	24.8 ± 3.2	22.2 ± 4.7	n.s.
Smoking	48	2	n.s.
Dyslipidemia	40	4	n.s.
Diabetes	54	5	n.s.
MI	6	2	n.s.
Revascularization	102	6	n.s.
Creatinine (mg/dl)	1.0 ± 0.3	1.3 ± 0.6	*p* < 0.01

Out of all the included nine clinical, eight magnetocardiographical (cf. Table 2), and nine newly introduced magnetoionographical (cf. Table 3) parameters we tested in this study, three parameters revealed themselves to be outstanding at predicting death: heart rate-corrected QT (QTc) prolongation, depression of repolarization current I_Kr_ + I_Ks_, and serum creatinine (all significant in Cox regression, Table 4, *p* < 0.05).

**Table 2:** Results of the MCG parameters in patients who survived the 6.5 years follow-up vs. the 11 deceased (Log_RMS3_ is the logarithm of RMS_3_).

	Survivors (*n* = 180)	Cardiac death (*n* = 11)	*p*-value
Heart Rate (bpm)	64.0 ± 11.5	75.7 ± 17.7	0.014
QRS (ms)	96.2 ± 19.6	99.6 ± 11.7	n.s.
T-Dispersion (ms)	9.3 ± 2.5	11.3 ± 2.5	0.014
QTc (ms)	391.2 ± 40.0	445.2 ± 55.8	0.001
RMS_1_ (pT)	3.4 ± 1.8	2.7 ± 1.2	n.s.
RMS_2_ (pT)	0.72 ± 0.67	0.51 ± 0.2	n.s.
RMS_3_ (pT)	1.3 ± 0.9	0.84 ± 0.3	0.037
RMS_4_ (pT)	1.02 ± 0.76	0.70 ± 0.2	n.s.
Log_RMS3_	0.05 ± 0.25	−0.11 ± 0.2	0.037

**Table 3:** Results of the MIG parameters in patients who survived the 6.5 years follow-up vs. the 11 deceased (Log_MI3_ is the logarithm of MI_3_).

	Survivors (*n* = 180)	Cardiac death (*n* = 11)	*p*-value
MI_1_ (pT)	1.76 ± 1.1	1.56 ± 0.91	n.s.
DI_1_ (pT)	1.7 ± 1.2	1.14 ± 0.46	n.s.
MI_2_ (pT)	0.37 ± 0.41	0.28 ± 0.17	n.s.
DI_2_ (pT)	0.34 ± 0.4	0.23 ± 0.17	n.s.
MI_3_ (pT)	0.53 ± 0.56	0.17 ± 0.11	0.001
DI_3_ (pT)	0.8 ± 0.66	0.67 ± 0.24	n.s.
MI_4_ (pT)	0.45 ± 0.44	0.23 ± 0.12	0.014
DI_4_ (pT)	0.57 ± 0.53	0.46 ± 0.2	n.s.
Log_MI3_	−0.43 ± 0.37	−0.84 ± 0.27	0.001
Ca uptake velocity (pT/s)	(−28.2) ± 24.2	(−20.1) ± 7.7	n.s.

**Table 4:** Significant parameters in the Cox regression model.

	B	SE	Wald	Df	Sig.	Exp(B)
Smoking	0.065	0.957	0.005	1	0.9459	0.937
Dyslipidemia	0.809	0.700	1.334	1	0.2480	0.445
DM	0.053	0.709	0.006	1	0.9404	0.948
Cr	1.265	0.613	4.263	1	**0** **.** **0390**	3.543
QTc	0.023	0.007	12.800	1	**0**.**0003**	1.024
LOG_MI3_	−3.757	1.262	8.868	1	**0**.**0029**	.023
Age	0.014	0.041	0.119	1	0.7296	1.014
Sex	−0.503	0.829	0.368	1	0.5442	1.654

Bold values represent *p* < 0.05.

**Figure 1:**
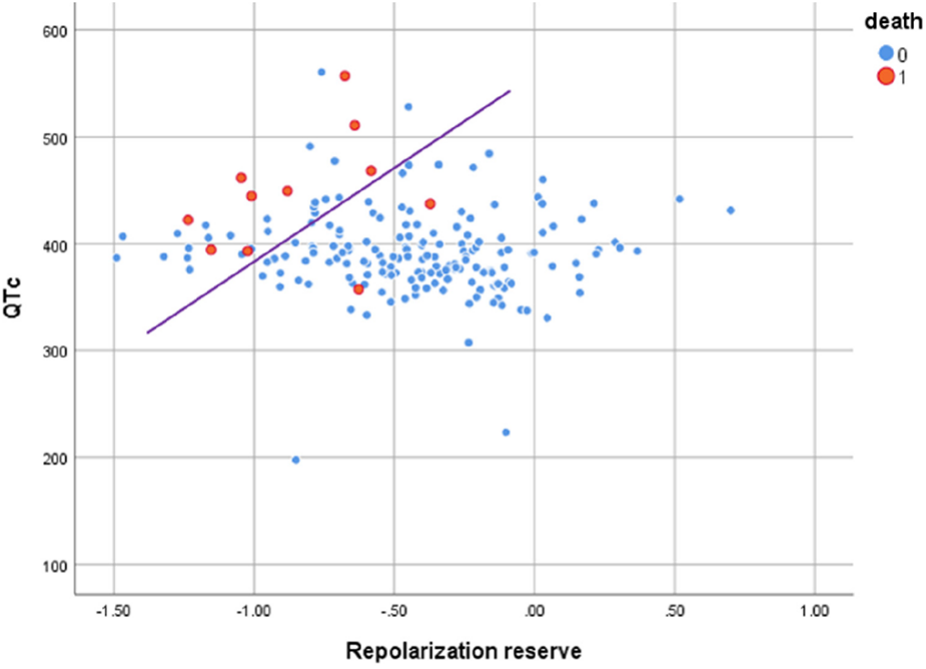
Scatter plot of QTc vs. repolarization reserve Log_MI3_ clearly separates the survivors and the cardiac deaths.

The best two parameters, QTc prolongation and the repolarization reserve Log_MI3_, demonstrate the classification potential in Figure 1. Almost all deceased (red) have higher QTc values and a decreased repolarization reserve (Log_MI3_). The negative predictive value is about 99% (the following cf. values).

**Figure 2:**
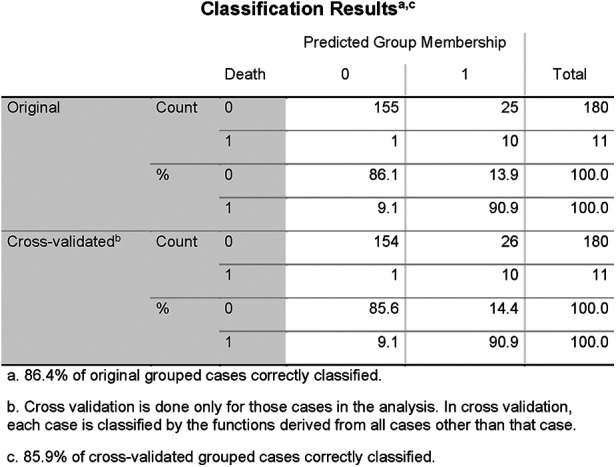
Classification results of linear discriminant function analysis using QTc, Log_MI3_, and Cr for the separation of the survivors (Group 0) and the patients who died of cardiac death (Group 1). Cross-validated sensitivity is 90.9% and specificity is 85.6%.

**Figure 3:**
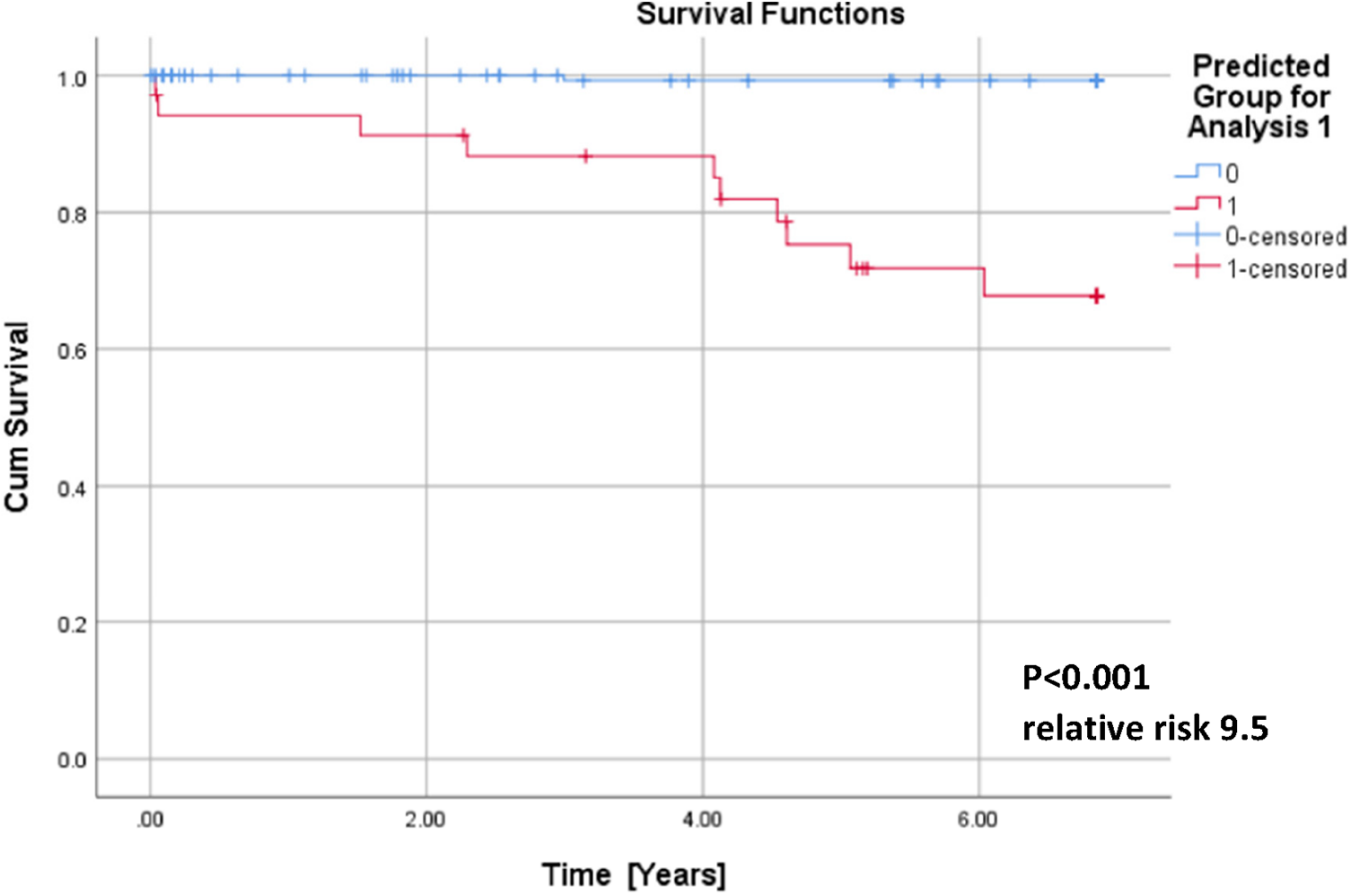
Cumulative survival functions of the Kaplan–Meier analysis using the three significant parameters from the Cox regression (Table 1) and predicted group memberships from Figure 2.

QTc, repolarization reserve, and creatinine clearly predicted cardiac death over the 6.5 years duration (Figure 2, sensitivity 90.9%, specificity 85.6%, negative predictive value 99.4%). Cardiac death risk was more than ninefold higher in patients with low repolarization reserve and QTc prolongation in comparison with the remaining ACP patients (Figure 3, *p* < 0.001). The non-parametric Kaplan–Meier statistics estimated significantly lower survival functions from their lifetime data (*p* < 0.001).

In a validation statistics, all nine newly introduced magnetoionographical parameters were left out of consideration. From the residual set of parameters, again QTc and Cr were automatically selected, instead of log_MI3_, log_RMS3_ was included. The discriminant function analysis led to a cross-validated sensitivity of 86.7%, a specificity of 81.1%, and a negative predictive value of 98.7% (the percentage of correctly classified persons is 86.4% and is similar to the previous discrimination).

## Discussion

This study investigated the potential of magnetocardiography at rest to predict mortality in patients with ACP. The main finding of our analysis was that QTc prolongation, increased serum creatinine, and a decreased repolarization reserve from MCG at rest are very sensitive and specific to predict mid-to-long-term mortality of ACP patients. The sensitivity of 90.9% for cardiac death, its specificity of 85.6%, and its outstanding negative predictive value of 99.4% offer new possibilities in clinical diagnostics. Our findings support the investigation of new prevention strategies and herald those new non-invasive techniques as complementary risk stratification tools—not just for patients with ACP.

It is well known that a prolongation of the QTc interval is a risk factor for cardiac death in the general population. Straus et al. investigated prospectively a population-based cohort of nearly 8,000 aging subjects (27). During the follow-up period of 6.7 years, 125 patients died because of sudden cardiac death. An abnormally prolonged QTc interval was associated with a threefold increased risk of sudden cardiac death (hazard ratio, 2.5; 95% confidence interval, 1.3–4.7), after adjustment for age, gender, body mass index, hypertension, cholesterol/high-density lipoprotein ratio, diabetes mellitus, myocardial infarction, heart failure, and heart rate. In patients with an age below the median of 68 years, the corresponding relative risk was 8.0 (95% confidence interval 2.1–31.3). Unfortunately, computer algorithms are unreliable in identifying prolonged QT, particularly in abnormal or poor-quality ECGs (28). Therefore, multichannel MCG measurements as used in this study should be favored as clinician screening tools owing to MCG’s ease of use and high accuracy. Accurately identifying patients with dangerously prolonged QT intervals allows clinicians to intervene on patients who are at acute risk of Torsade de Pointes and to avoid discharging patients at risk of sudden death.

Creatinine is a chemical compound left over from energy-producing processes. Healthy kidneys filter creatinine out of the blood. Possible causes of low creatinine levels are low muscle mass, liver problems, dietary factors, pregnancy, or a health condition. It is well known that low baseline serum creatinine concentrations increase the risk of mortality in critically ill patients (29). In patients with ACP, creatinine clearance is an important independent predictor of hospital death and major bleeding (30).

A decreased repolarization reserve (Log_MI3_) or T-wave amplitude (Log_RMS_) is known as a sensitive marker of SCD in high-risk hypertrophic cardiomyopathy and may provide incremental predictive value to established risk factors (31). Moreover, it was shown that low amplitude T-waves also are associated with SCD risk in the general population (32): Both negative T-waves and low amplitude T-waves are associated with cardiac death (HR 2.34; 95% CI 1.75–3.13 and HR 1.49; 95% CI 1.17–1.91, respectively) and death from any cause (HR 1.85; 95% CI 1.50–2.27 and HR 1.45; 95% CI 1.24–1.70, respectively). In the validation statistics of this investigation, the newly introduced MIG parameters were left out of consideration. Nevertheless, discriminant function analysis led to a cross-validated sensitivity of 86.7%, a specificity of 81.1%, and a negative predictive value of 98.7%, which is similar to the previous discrimination and underpins the stability of the results obtained. Our study supports the value of the concept of repolarization reserve that impairment of one type of transmembrane ion channel does not necessarily result in excessive repolarization changes because other repolarizing currents can take over and compensate. Patients with diminished repolarization reserve, however, are under increased risk of SCD (33). Therefore, reliably predictive non-invasive tests in clinical setting are sorely needed (33). The novel method of MIG as demonstrated in this study is capable of going beyond the assessment of QT interval alterations.

Roden proposed the widely accepted concept of “repolarization reserve,” the idea that the complexity of repolarization includes some redundancy (34). As a consequence, loss of one component (such as I_Kr_) ordinarily will not lead to failure of repolarization (i.e., marked QT prolongation); as a corollary, individuals with subclinical lesions in other components of the system, say I_Ks_ or calcium current, may display no QT change until I_Kr_ block is superimposed. Our recently developed MIG seems to characterize and quantify single membranous current as well as intracellular Ca^2+^ transient. Repolarization reserve would be, therefore, the sum of instantaneous total currents minus depolarization currents (ICa2+L, ICa2+T) and minus ICa2+transient in the time period of repolarization (QRS_end_–T_end_). Interestingly, repolarization currents flowing in the time period T_beg_–T_end_ (parameter MI3) were even more predictive than repolarization currents flowing in the time period QRS_end_–T_end_. This may be due to simultaneous flow of the early repolarization current I_to_, the transient outflow current.

MCG offers important practical advantages compared with other diagnostic methods: First, within 60 s measurement time it can detect the magnetic field of the heart in a contactless manner without exposing the patient to radiation. Second, it is less affected by conductivity variations caused by lungs, skin, and muscles compared with ECG. Technical and computational advances of MCG enable a fast and accessible screening with no negative effects for patients. However, the amplitude of the cardiac magnetic field decreases quadratically, and at greater distances cubically, with the distance of the sensor. It is therefore important to ensure that the distance between the sensor and the thorax is as small as possible and that this distance is recorded for later analysis (35). With the help of multichannel systems, the entire thoracic magnetic field can be assessed in one measurement (36). The retrospective nature of this investigation introduces some limitations: We cannot be sure that the results we obtained are too optimistic. Moreover, we need to prospectively show *in situ* that our MIG parameters actually represent the transmembrane ionic currents mentioned previously. Furthermore, we need to prospectively compare QTc and QT dispersion parameters calculated from both ECG and MCG. At this stage, we cannot prove that MCG provides additional information compared with ECG. However, we have backward compatibility for more than two decades in multicenter studies. Therefore, the application of innovative new algorithms to previous MCG records is prospective in nature. Moreover, a prospective validation study is ongoing: Survival data are currently being collected for approximately 2,000 MCG measurements from cardiac patients admitted to Yonsei University Hospital in the last decade.

Our data suggest structural and functional components to clinical life-threatening ventricular arrhythmogenesis. This approach will lead to a renaissance of MCG, which is a non-invasive, radiation-free tool for cardiac risk assessment and individualized treatment surveillance. Due to its high diagnostic accuracy and lack of any side-effects, MCG, in general, has the potential to become an ideal first-line diagnostics for all heart diseases.

## Data Availability

The raw data supporting the conclusions of this article will be made available by the authors, without undue reservation.
